# Hepatitis C virus genotype affects survival in patients with hepatocellular carcinoma

**DOI:** 10.1186/s12885-019-6040-3

**Published:** 2019-08-20

**Authors:** Hye Kyong Park, Sang Soo Lee, Chang Bin Im, Changjo Im, Ra Ri Cha, Wan Soo Kim, Hyun Chin Cho, Jae Min Lee, Hyun Jin Kim, Tae Hyo Kim, Woon Tae Jung, Ok-Jae Lee

**Affiliations:** 1 Department of Internal Medicine, Gyeongsang National University Changwon Hospital, Changwon, Republic of Korea; 2 Department of Internal Medicine, Gyeongsang National University School of Medicine and Gyeongsang National University Hospital, 15, Jinju-daero 816, Jinju, 52727 Republic of Korea; 3 Institute of Health Sciences, Gyeongsang National University, Jinju, Republic of Korea

**Keywords:** Hepatocellular carcinoma, Survival, Genotype, Hepatitis C virus

## Abstract

**Background:**

There is currently no evidence that hepatitis C virus (HCV) genotype affects survival in patients with hepatocellular carcinoma (HCC). This study aimed to investigate whether the HCV genotype affected the survival rate of patients with HCV-related HCC.

**Methods:**

We performed a retrospective cohort study using the data of patients with HCV-related HCC evaluated at two centers in Korea between January 2005 and December 2016. Propensity score matching between genotype 2 patients and non-genotype 2 patients was performed to reduce bias.

**Results:**

A total of 180 patients were enrolled. Of these, 86, 78, and 16 had genotype 1, genotype 2, and genotype 3 HCV-related HCC, respectively. The median age was 66.0 years, and the median overall survival was 28.6 months. In the entire cohort, patients with genotype 2 had a longer median overall survival (31.7 months) than patients with genotype 1 (28.7 months; *P* = 0.004) or genotype 3 (15.0 months; *P* = 0.003). In the propensity score–matched cohort, genotype 2 patients also showed a better survival rate than non-genotype 2 patients (*P* = 0.007). Genotype 2 patients also had a longer median decompensation-free survival than non-genotype 2 patients (*P* = 0.001). However, there was no significant difference in recurrence-free survival between genotype 2 and non-genotype 2 patients who underwent curative treatment (*P* = 0.077). In multivariate Cox regression analysis, non-genotype 2 (hazard ratio, 2.19; 95% confidence interval, 1.29–3.71) remained an independent risk factor for death.

**Conclusion:**

Among patients with HCV-related HCC, those with genotype 2 have better survival.

**Electronic supplementary material:**

The online version of this article (10.1186/s12885-019-6040-3) contains supplementary material, which is available to authorized users.

## Background

Hepatocellular carcinoma (HCC) is the sixth most prevalent cancer and the second leading cause of cancer-related mortality worldwide [1]. HCC is also the most common cause of death in patients with chronic hepatitis C virus (HCV) infection [2], with a median survival of 12–24 months [3–5]. The prevalence of HCV-related HCC varies by geographical region. HCV etiology is observed in approximately 30 and 50% of Asian and Caucasian HCC patients, respectively [6]. In Korea, which is among the endemic areas for hepatitis B virus (HBV) infection, approximately 13% of patients with HCC have HCV etiology [7, 8]. The prevalence of cirrhosis in patients with HCV-related HCC is approximately 80–90%; therefore, cirrhosis is the largest single risk factor for HCC development [9]. Among patients with HCV-related cirrhosis, the annual incidence of HCC is higher in Asian populations than in Western populations [10, 11].

The prognosis of HCC is affected by various factors such as tumor burden, underlying liver function, and patient performance status [12, 13]. The Barcelona Clinic Liver Cancer (BCLC) classification is now considered the best system for predicting survival in patients with HCC [13, 14]. However, additional factors not included in the BCLC system, such as alpha-fetoprotein (AFP) level, sex, ascites, total bilirubin, blood urea, prothrombin time-international normalized ratio (PT-INR), and Model For End-Stage Liver Disease (MELD) score, have also been demonstrated to have a prognostic value for predicting survival in HCC [3, 15–18]. The BCLC classification system, AFP, and the MELD score are considered to be correlated with tumor burden, tumor biology, and degree of liver function, respectively.

A meta-analysis of eight single-biopsy studies showed that there was a 50% increased rate of fibrosis progression in patients with HCV genotype 3 as compared to patients with other genotypes [19]. In addition, studies of liver graft reinfection by HCV demonstrated that the HCV genotype 1 was more frequently associated with progressive graft injury than the other genotypes [20, 21]. The HCV genotype affects the development of HCC in patients with chronic HCV infection. HCV genotype 1 infection in particular might play an important role in HCC development [22–24]. Recently, HCV genotype 3 infection has been emphasized to be associated with the possibility of HCC development [25–28]. Thus, an association between HCV genotypes 1 and 3 and the rapid progression of liver damage may result in poor survival in patients with HCV-related HCC.

Despite reports on the association between HCV genotype and disease severity of chronic hepatitis, evidence on the influence of the HCV genotype on the prognosis of patients with HCC is limited. Moreover, available data do not prove that the HCV genotype affects HCC survival [29–31]. To the best of our knowledge, no study has proven that the HCV genotype affects the survival of patients with HCV-related HCC. This study aimed to elucidate whether HCV genotypes affect the prognosis of patients with HCV-related HCC.

## Methods

### Study population

The cohort comprised patients consecutively diagnosed with a detectable genotype of HCV-related HCC at two centers from January 2005 to December 2016. The exclusion criteria were as follows: (1) a follow-up period of less than 6 months without death; (2) seropositivity for HBV surface antigen; and (3) seropositivity for the human immunodeficiency virus (HIV). The Institutional Review Boards of Gyeongsang National University Changwon Hospital and Gyeongsang National University Hospital approved this study.

### Data collection

The following laboratory test results were extracted from the medical records of the patients for analysis: HBV surface antigens, anti-HBV surface antibodies, anti-HCV, HCV RNA levels, HIV antibodies, AFP, serum albumin levels, aspartate aminotransferase, alanine aminotransferase levels, total bilirubin level, serum creatinine level, PT-INR, and platelet count. Comorbidities, including liver cirrhosis and diabetes mellitus, were also recorded. The patients’ medical and personal histories were carefully reviewed to identify age, sex, alcohol intake, antiviral treatment before and after enrollment, tumor characteristics such as the number and size of HCC nodules, the presence of vascular invasion and extrahepatic metastasis, and treatment modalities.

### Diagnosis and follow-up

The diagnosis of HCC was based on histological examination or typical radiographic findings, specifically, hepatic nodules with arterial enhancement and portal venous or delayed phase wash-out on contrast-enhanced computed tomography (CT) or magnetic resonance imaging (MRI) [32]. Liver cirrhosis was determined by liver biopsy or clinical, laboratory, and imaging findings. Heavy alcohol drinkers were defined as those who drank more than 60 g/day of alcohol. After diagnosis, all the patients underwent imaging examinations and laboratory tests every 3 months for a follow-up of disease status. Antiviral therapy using pegylated interferon alpha, ribavirin, and direct-acting agents were administered to the patients according to the clinical decisions of the treating physicians. Sustained virologic response (SVR) was defined as undetectable HCV RNA in the blood at 12 or 24 weeks after the end of antiviral treatment. To analyze tumor characteristics, the tumor stage (BCLC stage and modified Union for International Cancer Control (mUICC) TNM stage) [33, 34], Child-Pugh class, and MELD score were determined.

Treatment modalities for HCC during the study period were classified as surgical resection, radiofrequency ablation (RFA), chemoembolization (TACE), percutaneous ethanol injection (PEI), radiotherapy, systemic chemotherapy, sorafenib, and liver transplantation. Curative treatment modalities included hepatic resection, RFA, PEI, and transplantation. Recurrence-free survival was defined as the duration from the date of curative treatment to the date of local and/or distant recurrence or death. Hepatic decompensation was defined by the presence of ascites, hepatic encephalopathy, hepatorenal syndrome, or variceal hemorrhage as documented based on endoscopic examination. Time-to-event was calculated from the date of enrollment to the date of death, last observation, or December 31, 2018.

### Propensity score matching

The entire cohort was grouped according to HCV genotype (HCV genotypes 1, 2, and 3). We hypothesized that, among patients with HCV-related HCC, those with HCV genotype 2 have a better survival rate. Therefore, we performed propensity score matching to minimize the selection bias between genotype 2 and non-genotype 2 patients using the MatchIt package in R statistical software ver.3.1.3 (The R Foundation for Statistical Computing, Vienna, Austria). The propensity score was calculated from a logistic regression model that included age (years) and the presence of curative treatment modality for initial treatment.

### Statistical analysis

Continuous variables were expressed as the median (interquartile range). Intergroup differences in qualitative data were evaluated using the Fisher exact test, and the Mann-Whitney U test was used for quantitative data. Survival curves according to genotype and BCLC stage in the entire cohort and the propensity score–matched patients were calculated using the Kaplan–Meier method. Identified between-group differences were compared using the log-rank test. The association between HCV genotype 2 and survival was evaluated via univariate and multivariate analyses using the Cox proportional hazard model after adjusting for potential confounding variables. The risk was expressed as a hazard ratio (HR) and 95% confidence interval (CI). Statistical analyses were performed using PASW software (Version 18, SPSS Inc., Chicago, IL, USA), and a *P* value of < 0.05 was considered statistically significant.

## Results

### Patient characteristics

A total of 202 patients were identified, and 22 were excluded; therefore, 180 patients with HCV-related HCC were analyzed. Of these 180 patients, 86, 78, and 16 were infected with HCV genotypes 1, 2, and 3, respectively (Tables 1 and 2). The baseline characteristics of the 180 patients with HCV-related HCC are summarized in Table 1. The median age was 66.0 years, and HCC with HCV genotype 3 was diagnosed at a significantly younger age (median age, 46.0 years) than HCC with genotype 1 (64.5 years; *P* < 0.001) or genotype 2 (67.5 years; *P* < 0.001). The proportion of men was higher among genotype 3 patients (93.8%) than among genotype 2 patients (66.7%; *P* = 0.034). However, there was no significant difference in the rate of diabetes, cirrhosis, or alcohol consumption according to genotype.

**Table 1:** Baseline characteristics of the entire cohort according to genotype (*n* = 180)

	Genotype 1 (*n* = 86)	Genotype 2 (*n* = 78)	Genotype 3 (*n* = 16)
Age, year	64.5 (56.5–72.3)	67.5 (60.8–73.0) ^b^	46.0 (40.0–53.0) ^c^
Male gender	63 (73.3%)	52 (66.7%)^b^	15 (93.8%)
Biopsy for diagnosis	24 (27.9%)	27 (34.6%)	3 (18.8%)
Diabetes	27 (33.4%)	22 (34.9%)	6 (40.0%)
Cirrhosis	77 (89.5%)	68 (87.2%)	16 (100%)
Alcohol > 60 g/day	4 (4.7%)	4 (5.1%)	3 (18.8%)
SVR	15 (17.4%)	18 (23.1%)	2 (12.5%)
SVR before enrollment	5 (5.8%)	7 (9.0%)	0
SVR after enrollment	10 (11.6%)	11 (14.1%)	2 (12.5%)
HCV RNA > 600,000 *IU/mL*	44 (51.2%)	29 (37.2%)	6 (37.5%)
Creatinine, *mg/dL*	0.82 (0.70–0.93)	0.80 (0.70–0.92)	0.86 (0.69–0.99)
Bilirubin, *mg/dL*	0.99 (0.72–1.57)	1.00 (0.75–1.72)^b^	1.93 (1.32–3.67)^c^
Platelet, *× 10*^*9*^*/L*	111.5 (71.8–153.8)	105.5 (81.5–132.3)	86.5 (43.8–128.8)
Albumin, *g/dL*	3.6 (3.2–4.0)	3.5 (3.0–3.9)^b^	3.0 (2.7–3.6) ^c^
PT-INR	1.12 (1.04–1.20)	1.12 (1.06–1.25)^b^	1.35 (1.21–1.58) ^c^
Child Pugh B or C	16 (18.2%)	18 (23.1%)	10 (62.5%) ^c^
MELD score	8.0 (7.0–10.3)	9.0 (7.0–11.0) ^b^	12.5 (9.5–16.0) ^c^
Follow-up period (month)	28.7 (11.5–45.6)	31.7 (11.9–64.6) ^b^	15.0 (4.6–34.9)

*Abbreviation*: *HCV* hepatitis C virus, *PT-INR*, prothrombin time- international normalized ratio, *SVR* sustained virologic response, *MELD score* Model For End-Stage Liver Disease score

^a^ *p* < 0.05 genotype 1 vs genotype 2, ^b^ *p* < 0.05 genotype 2 vs genotype 3, ^c^ *p* < 0.05 genotype 1 vs genotype 3 using the Mann-Whitney U-test and Chi-squared test

Data are presented as the median (interquartile range) for continuous data and percentages for categorical data

**Table 2:** Tumor characteristics and treatment modalities of the entire cohort according to genotype (*n* = 180)

	Genotype 1 (*n* = 86)	Genotype 2 (*n* = 78)	Genotype 3 (*n* = 16)
AFP, *ng/mL*	19.9 (9.3–93.2)	41.8 (8.4–100.2) ^b^	21.7 (7.3–72.9)
Within Milan criteria	53 (61.6%)	52 (66.7%)	8 (50.0%)
Malignant vascular invasion	7 (8.1%)	4 (5.1%) ^b^	4 (25.0%)
Extrahepatic metastasis	3 (3.5%)	1 (1.3%)	2 (12.5%)
HCC nodules			
1	47 (54.7%)	48 (61.5%)	5 (31.3%)
2~3	19 (22.1%)	21 (26.9%)	6 (37.5%)
≥ 4	20 (23.3%)	9 (11.5%)	5 (31.3%)
Largest tumor size			
< 2 cm	27 (30.7%)	21 (26.9%)	6 (37.5%)
2 ~ 5 cm	41 (47.7%)	45 (57.7%)	5 (31.3%)
> 5 cm	18 (20.9%)	12 (15.4%)	5 (31.3%)
BCLC ^b c^			
0	15 (17.4%)	10 (12.8%)	1 (6.3%)
A	41 (47.7%)	46 (59.0%)	7 (43.8%)
B	19 (22.1%)	14 (17.9%)	1 (6.3%)
C	10 (11.6%)	5 (6.4%)	5 (31.3%)
D	1 (1.2%)	3 (3.8%)	2 (12.5%)
mUICC ^b c^			
1	15 (17.4%)	13 (16.7%)	2 (12.5%)
2	39 (45.3%)	39 (50.0%)	8 (50.0%)
3	22 (25.6%)	24 (30.8%)	4 (25.0%)
4	10 (11.6%)	2 (2.6%)	2 (12.5%)
Treatment modality			
Resection	17 (19.8%)	23 (29.5%)	1 (6.3%)
RFA	23 (26.7%)	19 (24.4%) ^b^	0 ^c^
TACE	58 (67.4%) ^a^	40 (51.3%)	11 (68.8%)
PEI	1 (1.2%)	1 (1.3%)	0
Radiotherapy	10 (11.6%)	6 (7.7%)	1 (6.3%)
Systemic chemotherapy	2 (2.3%)	1 (1.3%)	0
Sorafenib	4 (4.7%)	1 (1.3%)	1 (6.3%)
Liver transplantation	0	1 (1.3%)	0
No Treatment	9 (10.5%)	12 (15.4%)	2 (12.5%)
Curative Treatment (Initial)	26 (33.7%)	36 (46.2%) ^b^	1 (6.3%) ^c^
Recurrence after curative treatment (*n* = 66)	20 (69.0%)	21 (58.3%)	1 (100%)
Decompensation	47 (54.7%) ^a^	22 (28.6%) ^b^	13 (81.3%)
Death	49 (57.0%) ^a^	29 (37.2%) ^b^	11 (68.8%)

*Abbreviation*: *AFP* Alpha-fetoprotein, *BCLC* Barcelona Clinic Liver Cancer, *HCC* Hepatocellular carcinoma, *RFA* Radiofrequency ablation, *TACE*, Transarterial chemoembolization, *PEI* Percutaneous ethanol injection

^a^ *p* < 0.05 genotype 1 vs genotype 2, ^b^ *p* < 0.05 genotype 2 vs genotype 3, ^c^ *p* < 0.05 genotype 1 vs genotype 3 using the Mann-Whitney U-test and Chi-squared test

Data are presented as the median (interquartile range) for continuous data and percentages for categorical data

In laboratory tests, patients with genotype 3 had higher bilirubin and PT-INR levels but lower albumin levels than patients with genotype 1 and genotype 2. Liver function as assessed according to the MELD score and Child-Pugh class was worse in genotype 3 patients than genotype 1 and genotype 2 patients. Of the 180 patients in the entire cohort, 12 achieved SVR before enrollment, whereas 23 achieved SVR after enrollment (Table 1).

### Tumor characteristics and tumor stage

The tumor characteristics and treatment modalities of the entire cohort are summarized in Table 2. Patients with genotype 2 exhibited higher AFP levels than those with genotype 3. Additionally, patients with genotype 2 presented with a significantly lower rate of malignant vascular invasion than those with genotype 3. However, there were no significant differences in Milan criteria, extrahepatic invasion, HCC nodules, and largest tumor size according to genotype. However, tumor stage, as measured by the BCLC and mUICC classifications, was worse in genotype 3 patients than in genotype 1 and genotype 2 patients.

### Treatment modalities and overall survival in the entire cohort

Regarding treatment modalities for HCC, the proportion of genotype 3 patients who underwent RFA (0%) was lower than that in genotype 1 (26.7%; *P* = 0.020) and genotype 2 (24.4%; *P* = 0.036) patients. The proportion of genotype 2 patients who underwent TACE (51.3%) was lower than that of genotype 1 patients (67.4%, *P* = 0.039). No significant differences were noted in other treatment modalities, including hepatic resection, PEI, systemic chemotherapy, liver transplantation, and no treatment. The proportion of genotype 3 patients administered a curative treatment modality for initial treatment (6.3%) was lower than that of genotype 1 (33.7%; *P* = 0.034) and genotype 2 (46.2%; *P* = 0.004) patients.

**Fig. 1:**
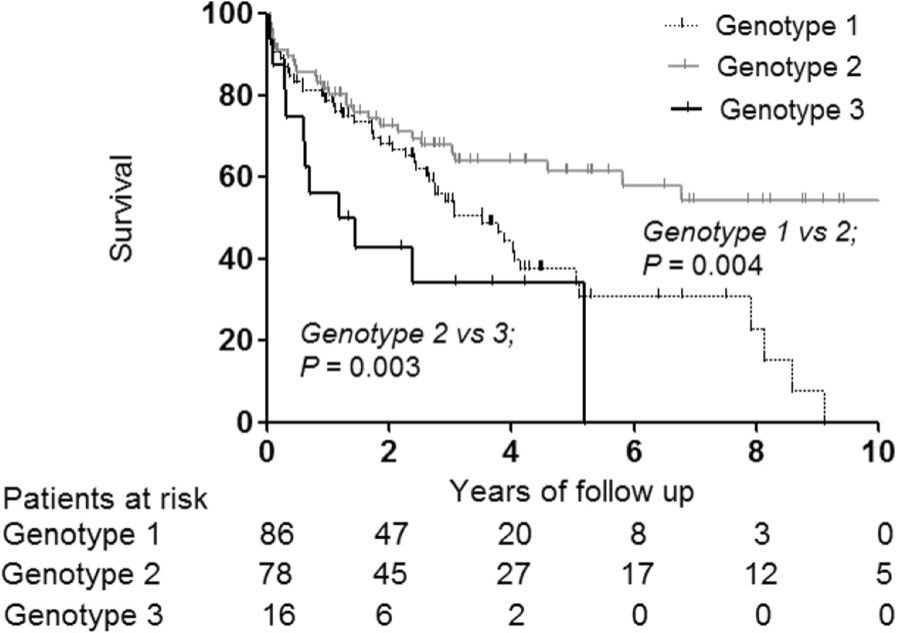
Overall survival according to HCV genotype in the entire cohort (*n* = 180). HCV: hepatitis C virus

The median overall survival was 28.6 months (interquartile range, 11.1–50.2 months), and the 5-year overall survival rate was 47.5%. During the study period, the mortality rate among genotype 2 patients (*n* = 29, 37.2%) was lower than that among genotype 1 (*n* = 49, 57.0%; *P* = 0.013) and genotype 3 (*n* = 11, 68.8%; *P* = 0.027) patients (Table 2). The 12-month survival rates for patients with BCLC stage 0, A, B, C, and D disease were 92.3, 92.4, 63.7, 30.0, and 16.7%, respectively (Additional file 1: Figure S1). In the entire cohort, patients with genotype 2 had longer overall survival than patients with genotype 1 (*P* = 0.004) and genotype 3 (*P* = 0.003) (Fig. 1).

### Survival analysis in propensity score–matched patients

**Fig. 2:**
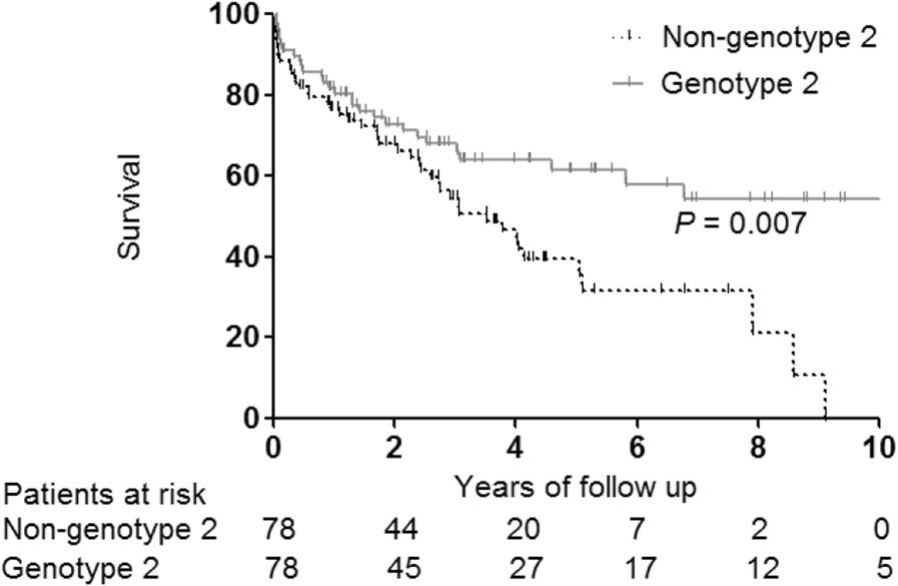
Overall survival according to HCV genotype in propensity score–matched patients (*n* = 156). HCV: hepatitis C virus

After calculating the propensity score, 78 pairs of patients in the genotype 2 group and the non-genotype 2 group were matched using a 1:1 nearest neighbor matching algorithm (Additional file 2: Figure S2). The baseline characteristics and tumor characteristics for the matched groups are listed in Additional file 3: Tables S1 and S2. Patients with genotype 3 had the worst survival in the entire cohort, probably because they might not have achieved a curative treatment due to their poor liver function and advanced tumor stage at baseline compared to other genotypes. Therefore, to minimize the selection bias for the occurrence of mortality in this study, further analyses were performed in propensity score–matched patients. No significant differences in baseline characteristics (Additional file 3: Table S1) or tumor characteristics and treatment modalities (Additional file 3: Table S2) were noted between matched genotype 2 and non-genotype 2 patients. However, the genotype 2 group had longer overall survival than the non-genotype 2 group (*P* = 0.007) (Fig. 2).

Univariate analysis showed that non-genotype 2, AFP > 200 ng/mL, MELD score per point, Child-Pugh class B or C, SVR, and BCLC stage were related to mortality (Table 3). On multivariate analysis, the independent factors for death were non-genotype 2 (HR, 2.19; 95% CI, 1.29–3.71); MELD score per point (HR, 1.23; 95% CI, 1.11–1.37); SVR (HR, 0.18; 95% CI, 0.06–0.52); and BCLC stage A (HR, 3.32; 95% CI, 1.20–9.19), stage B (HR, 6.06; 95% CI, 2.08–17.69), stage C (HR, 18.83; 95% CI, 6.06–58.52), and stage D (HR, 8.87; 95% CI, 2.02–39.02).

**Table 3:** Univariate and multivariate analyses showing significant predictive factors of mortality in the propensity score–matched patients (*n* = 156)

Variable	Univariate analysis		Multivariate analysis	
	*P*	HR (95% CI)	*P*	HR (95% CI)
Non-genotype 2	0.007	1.93 (1.19–3.13)	0.004	2.19 (1.29–3.71)
AFP > 200 *ng/mL*	< 0.001	3.11 (1.86–5.20)	0.001	2.93 (1.55–5.55)
MELD score per point	< 0.001	1.15 (1.08–1.23)	< 0.001	1.23 (1.11–1.37)
Child Pugh class B or C	< 0.001	2.71 (1.66–4.54)	0.287	0.67 (0.31–1.41)
SVR	< 0.001	0.150 (0.06–0.41)	0.002	0.18 (0.06–0.52)
BCLC Stage 0	Reference		Reference	
Stage A	0.235	1.78 (0.69–4.59)	0.021	3.32 (1.20–9.19)
Stage B	0.001	5.30 (1.97–14.38)	0.001	6.06 (2.08–17.69)
Stage C	< 0.001	13.16 (4.70–36.87)	< 0.001	18.83 (6.06–58.52)
Stage D	< 0.001	25.08 (6.45–97.56)	0.004	8.87 (2.02–39.02)

*Abbreviation*: *HR* hazard ratio, *CI* confidence interval, *AFP* alpha-fetoprotein, *SVR* sustained virologic response, *BCLC* Barcelona Clinic Liver Cancer

**Fig. 3:**
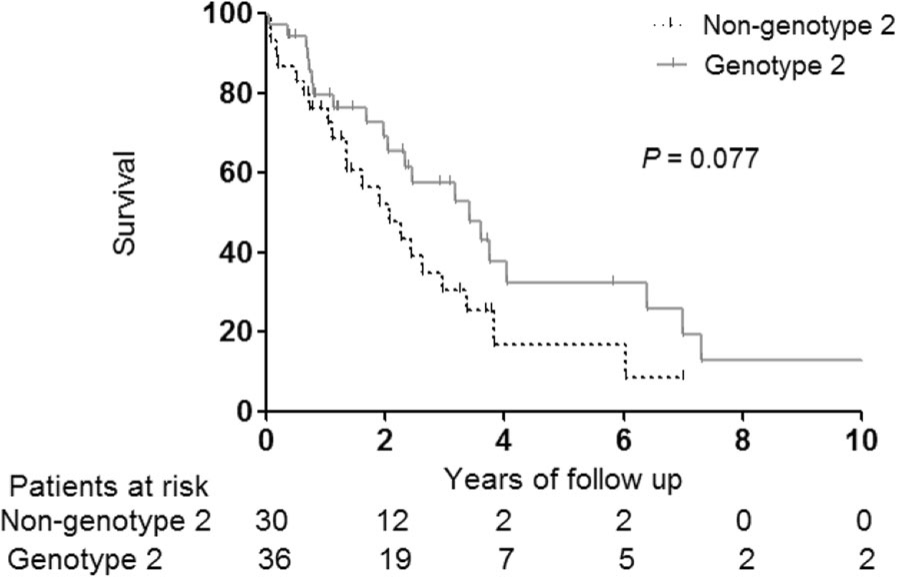
Recurrence-free survival in patients who underwent curative treatment of HCC in the propensity score–matched groups (*n* = 66). HCC: hepatocellular carcinoma

**Fig. 4:**
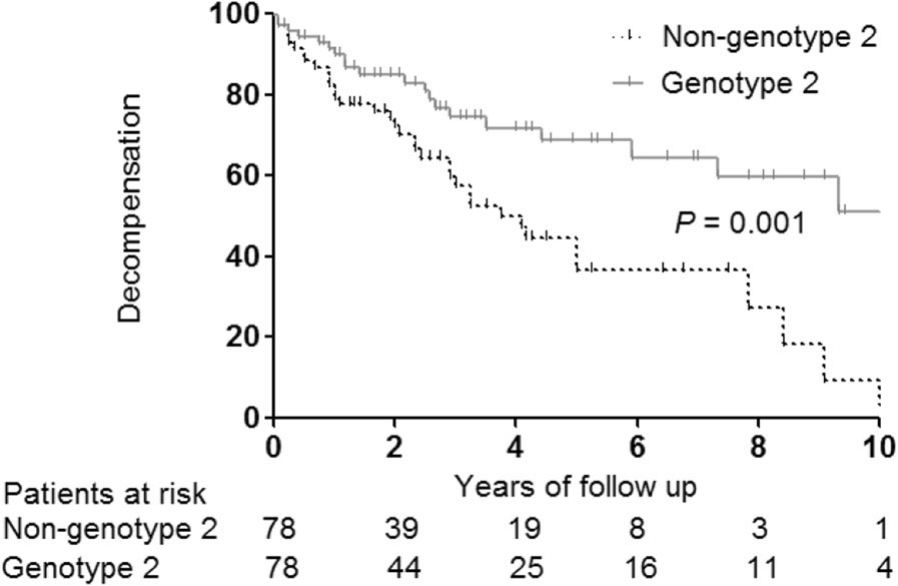
Decompensation-free survival according to HCV genotype in propensity score-matched patients (*n* = 156). HCV: hepatitis C virus

Regarding the recurrence-free survival of 66 patients who underwent curative treatment for initial treatment, no significant differences between the genotype 2 and non-genotype 2 groups (*P* = 0.077) (Fig. 3) were noted. In patients with HCC who received curative treatment, the 5-year survival rate was 76.7%. In 156 propensity score-matched patients, the decompensation-free survival was longer in patients with genotype 2 than in those with other genotypes (*P* = 0.001) (Fig. 4).

## Discussion

This multicenter, retrospective, observational study involving patients with HCV-related HCC in Korea showed that HCV genotype affects the survival of patients of HCC. In the entire cohort, patients with genotype 2 had longer overall survival than patients with other genotypes. In the propensity score–matched cohort, patients with genotype 2 also had a better survival rate than non-genotype 2 patients. On multivariate analysis, non-genotype 2 remained an independent risk factor for death (HR: 2.19). The decompensation-free survival was longer in patients with genotype 2 than in those with other genotypes. However, there was no significant difference in recurrence-free survival between genotype 2 and non-genotype 2 patients who underwent curative treatment.

A previous meta-analysis of observational studies of HCC reported that the rates of any treatment and curative treatment were 53 and 22%, respectively [35]. In the subgroup analysis of early HCC, the curative treatment rate was 59%. In the current study, 87.2% of patients in the entire cohort received any treatment and 36.7% received curative treatment. This suggests that our patients were more actively treated for HCC than patients in previous studies. The median overall survival was 28.6 months, and the 5-year overall survival rate was 47.5%, which are similar to those observed in previous studies of Asian patients [36, 37]. In previous studies [38–40], the 5-year survival rate associated with the curative treatment of patients with early HCC was 50–70%, which is lower than our results (76.7%).

Although there have been some studies on the relationship between HCV genotype and survival in HCC, there is no report that genotype affects the survival rate of HCC. Toyoda et al. compared the outcomes of small HCC lesions (≤2 cm in diameter) in patients with HCV genotype 1 and genotype 2 and reported no differences in either survival or overall recurrence rate according to genotype. However, they found that genotype 2 patients showed a significantly higher rate of intrahepatic metastasis than non-genotype 2 patients [30]. Shindoh et al. reported that the HCV genotype was not correlated with either the overall survival or tumor recurrence rate in 199 patients who underwent curative liver resection for HCV-related HCC. Akamatsu et al. reported that the HCV genotype did not affect either the survival or recurrence rates in a cohort of 307 patients with HCV-related HCC [29]. However, all of these studies are limited to the Japanese population. In addition, only the study by Akamatsu et al. included patients with all stages of HCC.

To the best of our knowledge, our study is the first to report that the HCV genotype affects the survival of patients with HCV-related HCC. Particularly, HCC patients with HCV genotype 2 showed better survival. Moreover, our study used propensity score matching to minimize selection bias between genotype 2 and non-genotype 2 patients. In patients who received curative treatment, patients with genotype 2 tended to show a better recurrence-free survival rate than non-genotype 2 patients, although this difference was not statistically significant (*P* = 0.077). However, a better decompensation-free survival rate was observed in patients with genotype 2 than in those with other genotypes. These results suggest that the HCV genotype affects the degree of liver function rather than the tumor biology, thereby affecting the overall survival. Traditionally, HCV genotype 1 has been reported to be associated with more severe liver disease and a more aggressive course than other genotypes [41]. HCV genotype 1 in patients undergoing liver transplantation is associated with earlier recurrence and more severe hepatitis than other genotypes [20, 21]. Furthermore, a possible association of genotype 1 with HCC has been proposed [22–24]. More recent studies reported that HCV genotype 3 is more closely associated with the risk of developing end-stage liver disease and HCC than other genotypes [26–28]. These studies support the possibility that HCV genotypes 1 and 3 may adversely affect survival after sustained negative effects on liver function even after HCC development. Traditionally, HCV genotype 1 is an independent factor for HCC through mechanisms of chronic inflammation, liver cell necrosis, and extensive fibrosis [42–44]. The mechanisms underlying the aggressiveness of HCV genotype 3 are not well known. However, hepatic steatosis, accelerated fibrosis, and insulin resistance observed in HCV genotype 3 infection may contribute towards poor prognosis [45]. In our previous study on patients infected with HCV without HCC, we reported that genotype 3 was an independent factor for HCC and liver-related mortality [28]. In addition, the genotype 3 infection was the most aggressive infection in this study of HCC patients.

Surprisingly, univariate analysis showed that HCV RNA level (> 600,000 IU/mL) was not associated with survival in patients with HCC (*P* = 0.354, 95% CI = 0.50–1.29), which is similar to the findings of previous studies [27, 29]. However, this result was in contrast to previous reports that higher levels of HBV DNA in patients with chronic HBV infection increase the risk of HCC and cirrhosis [46, 47].

There were a few limitations associated with our study. First, all participants were Korean. However, our study included the three most common HCV genotypes. Second, our study was limited by the retrospective nature of its design. Although the baseline factors were well matched in propensity-score matching, imbalances (although not statistically significant) were observed in the proportion of curative treatments in treatment modalities between non-genotype 2 and genotype 2 (35.9% vs. 46.2%). Multicenter prospective studies will be needed in the future to confirm whether HCV genotype affects the survival rate of patients with HCC.

## Conclusion

Among patients with HCV-related HCC treated with various modalities, including curative, non-curative, and supportive treatment, patients with HCV genotype 2 had longer overall survival than those with other genotypes. Our results suggest that the HCV genotype affects overall survival by influencing the liver function.

## Additional files

Additional file 1: **Figure S1.** Kaplan-Meier curve showing overall mortality in the entire cohort stratified by BCLC stage (*n* = 180). BCLC: Barcelona Clinic Liver Cancer (TIF 2018 kb)

Additional file 2: **Figure S2.** Patient recruitment flow chart. (TIF 5176 kb)

Additional file 3: **Table S1.** Baseline characteristics of the propensity score–matched patients (*n* = 156). **Table S2.** Tumor characteristics and treatment modalities of the propensity score–matched patients (n = 156). (DOCX 28 kb)

## Data Availability

The datasets used and/or analyzed during the current study are available from the corresponding author on reasonable request.

## Abbreviations

AFP: Alpha-fetoprotein
BCLC: Barcelona Clinic Liver Cancer
CI: Confidence interval
CT: Computed tomography
HBV: Hepatitis B virus
HCC: Hepatocellular carcinoma
HCV: Hepatitis C virus
HIV: Human immunodeficiency virus
HR: Hazard ratio
MELD: Model For End-Stage Liver Disease
MRI: Magnetic resonance imaging
mUICC: Modified Union for International Cancer Control
PEI: Percutaneous ethanol injection
PT-INR: Prothrombin time- international normalized ratio
RFA: Radiofrequency ablation
SVR: Sustained virologic response
TACE: Transarterial chemoembolization
