# Supplementary figures and images for: Hepatitis C virus genotype affects survival in patients with hepatocellular carcinoma
Source: BMC Cancer. 2019 Aug 20;19:822. doi: 10.1186/s12885-019-6040-3 (PMC6700836; doi:10.1186/s12885-019-6040-3)

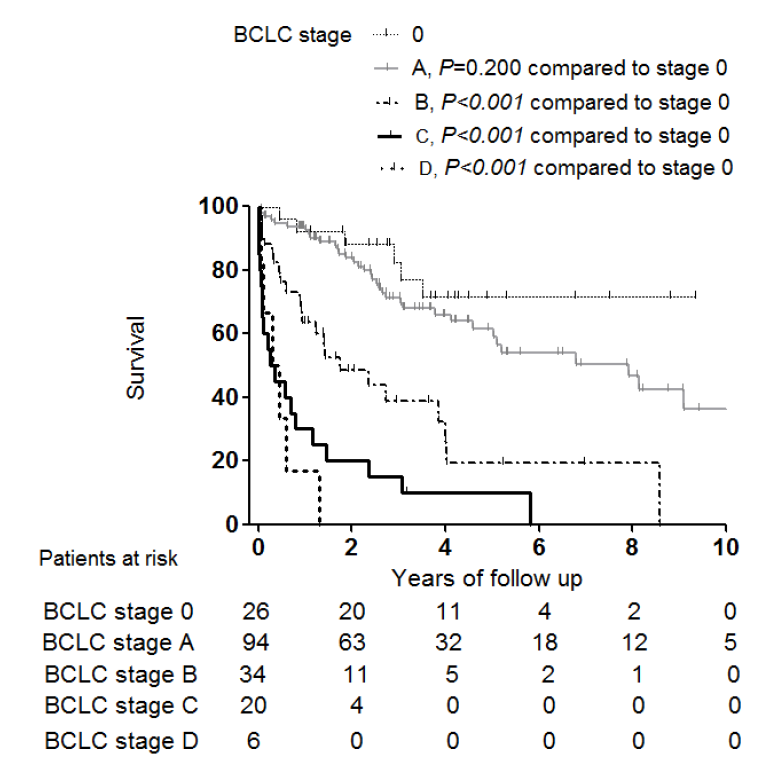

Supplement: Supplementary file 1 — Figure S1. Kaplan-Meier curve showing overall mortality in the entire cohort stratified by BCLC stage (n = 180). BCLC: Barcelona Clinic Liver Cancer (TIF 2018 kb) [file 12885_2019_6040_MOESM1_ESM.tif]

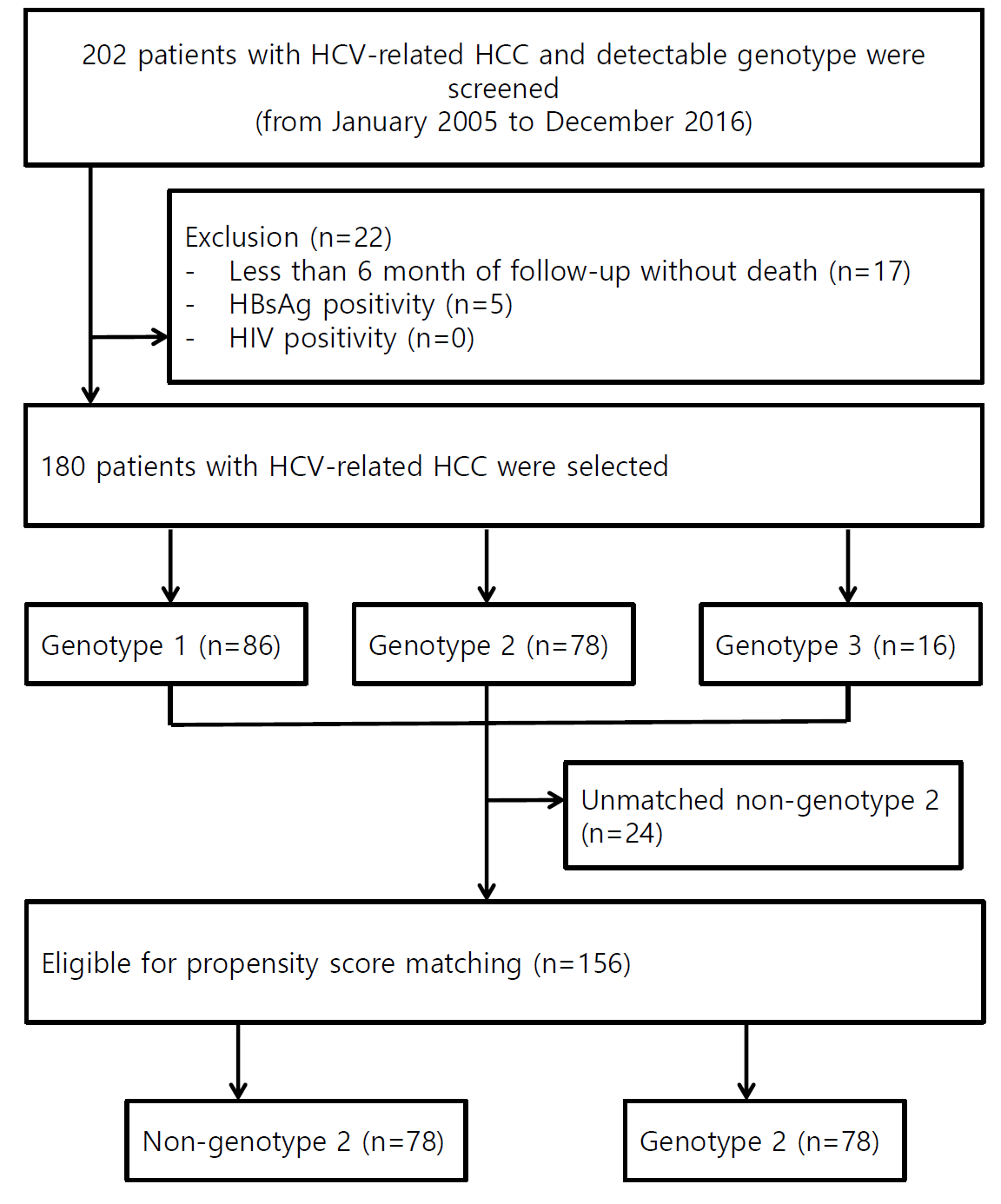

Supplement: Supplementary file 2 — Figure S2. Patient recruitment flow chart. (TIF 5176 kb) [file 12885_2019_6040_MOESM2_ESM.tif]
