# Supplementary material for: Hepatitis C virus genotype affects survival in patients with hepatocellular carcinoma
Source: BMC Cancer. 2019 Aug 20;19:822. doi: 10.1186/s12885-019-6040-3 (PMC6700836; doi:10.1186/s12885-019-6040-3)
Supplement: Supplementary file 3 — Table S1. Baseline characteristics of the propensity score–matched patients (n = 156). Table S2. Tumor characteristics and treatment modalities of the propensity score–matched patients (n = 156). (DOCX 28 kb) [file 12885_2019_6040_MOESM3_ESM.docx]

Additional file 1: Table S1. Baseline characteristics of the propensity score–matched patients (n=156)

|  | Non-genotype 2 (n=78) | Genotype 2 (n=78) | *P* |
| --- | --- | --- | --- |
| Age, year | 66.0 (58.0-73.3) | 67.5 (60.8-73.0) | 0.253 |
| Male gender | 56 (71.8%) | 52 (66.7%) | 0.603 |
| Diabetes | 22 (28.2%) | 22 (34.9%) | 0.465 |
| Cirrhosis | 69 (88.5%) | 68 (87.2%) | 1.000 |
| Alcohol > 60 g/day | 2 (2.6%) | 4 (5.1%) | 0.681 |
| SVR | 13 (16.7%) | 18 (23.1%) | 0.423 |
| SVR before enrollment | 4 (5.1%) | 8 (10.3%) | 0.368 |
| SVR after enrollment | 9 (11.5%) | 11 (14.1%) | 0.811 |
| HCV RNA > 600,000 *IU/mL* | 38 (48.7%) | 29 (37.2%) | 0.196 |
| Creatinine, *mg/dL* | 0.83 (0.70-0.93) | 0.80 (0.70-0.92) | 0.338 |
| Bilirubin, *mg/dL* | 1.00 (0.72-1.57) | 1.00 (0.75-1.72) | 0.689 |
| Platelet, *×10^9^/L* | 118.0 (77.5-158.3) | 105.5 (81.5-132.3) | 0.356 |
| Albumin, *g/dL* | 3.6 (3.1-4.1) | 3.5 (3.0-3.9) | 0.260 |
| PT-INR | 1.12 (1.03-1.20) | 1.12 (1.06-1.25) | 0.253 |
| Child Pugh B or C | 16 (20.5%) | 18 (23.1%) | 0.846 |
| MELD score | 8.0 (7.0-11.0) | 9.0 (7.0-11.0) | 0.640 |
| Follow-up period (month) | 28.9 (10.8-48.1) | 31.7 (11.9-64.6) | 0.155 |
| Genotype |  |  |  |
| Genotype 1 | 73 (46.8%) |  |  |
| Genotype 2 |  | 78 (50%) |  |
| Genotype 3 | 5 (3.2%) |  |  |

Abbreviation: PT-INR, prothrombin time- international normalized ratio; SVR, sustained virologic response; MELD score, Model For End-Stage Liver Disease score.

^a^ *p* < 0.05 genotype 1 vs genotype 2, ^b^ *p* < 0.05 genotype 2 vs genotype 3, ^c^ *p* < 0.05 genotype 1 vs genotype 3 using the Mann-Whitney U-test and Chi-squared test.

Data are presented as the median (interquartile range) for continuous data and percentages for categorical data.

Additional file 2: Table S2. Tumor characteristics and treatment modalities of the propensity score–matched patients (n=156)

|  | Non-genotype 2 (n=78) | | Genotype 2 (n=78) | | *P* |
| --- | --- | --- | --- | --- | --- |
| AFP, *ng/mL* | 24.2 (9.9-114.5) | | 41.8 (8.4-100.2) | | 0.332 |
| Within Milan criteria | 47 (60.3%) | | 52 (66.7%) | | 0.506 |
| Malignant vascular invasion | 6 (7.7%) | | 4 (5.1%) | | 0.746 |
| Extrahepatic metastasis | 2 (2.6%) | | 1 (1.3%) | | 1.000 |
| HCC nodules |  | |  | | 0.339 |
| 1 | 43 (55.1%) | | 48 (61.5%) | |  |
| 2~3 | 16 (20.5%) | | 21 (26.9%) | |  |
| ≥4 | 19 (24.4%) | | 9 (11.5%) | |  |
| Largest tumor size |  | |  | | 0.570 |
| <2cm | 21 (26.9%) | | 21 (26.9%) | |  |
| 2 ~ 5cm | 40 (51.3%) | | 45 (57.7%) | |  |
| >5cm | 17 (21.8%) | | 12 (15.4%) | |  |
| BCLC |  | |  | | 0.420 |
| 0 | 12 (15.4%) | | 10 (12.8%) | |  |
| A | 38 (48.7%) | | 46 (59.0%) | |  |
| B | 17 (21.8%) | | 14 (17.9%) | |  |
| C | 10 (12.8%) | | 5 (6.4%) | |  |
| D | 1 (1.3%) | | 3 (3.8%) | |  |
| mUICC |  | |  | | 0.270 |
| 1 | 13 (16.7%) | | 13 (16.7%) | |  |
| 2 | | 36 (46.2%) | | 39 (50.0%) |  |
| 3 | | 21 (26.9%) | | 24 (30.8%) |  |
| 4 | | 8 (10.3%) | | 2 (2.6%) |  |
| Treatment modality | |  | |  |  |
| Resection | | 18 (23.1%) | | 23 (29.5%) | 0.467 |
| RFA | | 18 (23.1%) | | 19 (24.4%) | 1.000 |
| TACE | | 50 (64.1%) | | 40 (51.3%) | 0.144 |
| PEI | | 1 (1.3%) | | 1 (1.3%) | 1.000 |
| Radiotherapy | | 9 (11.5%) | | 6 (7.7%) | 0.588 |
| Systemic chemotherapy | | 2 (2.6%) | | 1 (1.3%) | 1.000 |
| Sorafenib | | 4 (5.1%) | | 1 (1.3%) | 0.367 |
| Liver transplantation | | 0 | | 1 (1.3%) | 1.000 |
| No Treatment | | 8 (10.3%) | | 12 (15.4%) | 0.473 |
| Curative Treatment (Initial) | | 28 (35.9%) | | 36 (46.2%) | 0.254 |

Abbreviation: AFP, Alpha-fetoprotein; BCLC, Barcelona Clinic Liver Cancer; HCC, Hepatocellular carcinoma; RFA, Radiofrequency ablation; TACE, Transarterial chemoembolization; PEI, Percutaneous ethanol injection.

^a^ *p* < 0.05 genotype 1 vs genotype 2, ^b^ *p* < 0.05 genotype 2 vs genotype 3, ^c^ *p* < 0.05 genotype 1 vs genotype 3 using the Mann-Whitney U-test and Chi-squared test.

Data are presented as the median (interquartile range) for continuous data and percentages for categorical data.
